# Estimating the Clinical and Economic Impact of Switching from the 13-Valent Pneumococcal Conjugate Vaccine (PCV13) to Higher-Valent Options in Greek Infants

**DOI:** 10.3390/vaccines11081369

**Published:** 2023-08-15

**Authors:** Sophie Warren, Myrto Barmpouni, Vasiliki Kossyvaki, George Gourzoulidis, Johnna Perdrizet

**Affiliations:** 1 Global Health Economic and Outcomes Research, Pfizer Inc., New York, NY 10001, USA; 2 Pfizer Hellas, 154 51 Athens, Greece; 3 Health Through Evidence, 174 56 Athens, Greece; 4 Global Health Economics and Outcomes Research, Pfizer Canada, Kirkland, QC H9J 2M5, Canada

**Keywords:** pneumococcal disease, pneumococcal conjugate vaccines, PCV, invasive pneumococcal disease, cost-effectiveness, otitis media, pneumonia

## Abstract

In June 2010, Greece introduced the 13-valent pneumococcal conjugate vaccine (PCV13) for pediatric vaccination and has since observed a large decrease in pneumococcal disease caused by these vaccine serotypes, yet the disease prevalence of non-vaccine serotypes has increased. Two higher-valent conjugate vaccines, a 15-valent (PCV15) and a 20-valent (PCV20), were developed to improve serotype coverage and combat serotype replacement. A decision-analytic model was adapted to the Greek setting using historical pneumococcal disease trends from PCV13 to forecast future clinical and economic outcomes of higher-valent PCVs over a 10-year period (2023–2033). The model estimated outcomes related to invasive pneumococcal disease (IPD), hospitalized and non-hospitalized pneumonia, and otitis media (OM) resulting from a switch in vaccination programs to PCV15 in 2023 or switching to PCV20 in 2024. Cost-effectiveness was evaluated from the third-party payer’s perspective in the Greek healthcare system. Compared to implementing PCV15 one year earlier, switching from PCV13 to PCV20 in 2024 was estimated to be a cost-saving strategy by saving the Greek health system over EUR 50 million in direct medical costs and averting over 250 IPD cases, 54,800 OM cases, 8450 pneumonia cases, and 255 deaths across all ages over a 10-year period.

## 1. Introduction

*Streptococcus pneumoniae* is a Gram-positive bacterium that asymptomatically colonizes the upper respiratory tract and is a leading cause of lower respiratory infection morbidity and mortality globally [1,2]. Pneumococcal disease remains one of the most common vaccine-preventable diseases worldwide and manifests clinically as invasive pneumococcal disease (IPD), such as bacteremia, meningitis, sepsis, and bacteremic pneumonia. More commonly, pneumococcal disease presents as a non-invasive mucosal disease, such as pneumonia and otitis media (OM). Pediatric IPD and non-invasive disease are associated with significant clinical and economic burden in the form of both direct and indirect costs [3,4].

Vaccination is the most effective strategy for preventing pneumococcal disease, and the introduction of pneumococcal conjugate vaccines (PCVs) in pediatric national immunization programs (NIPs) has resulted in significant impact against vaccine serotypes from widespread use [5]. Countries throughout Europe have observed substantial declines in IPD, pneumonia, and OM incidence in both vaccinated and unvaccinated populations following the introduction of the 13-valent pneumococcal conjugate vaccine (PCV13) [6,7,8,9,10]. In Greece, PCV13 was included in the pediatric NIP as of June 2010 in a 3 + 1 dosing schedule, with PCV13 catch-up vaccination also recommended in the NIP for all children aged less than 60 months and previously vaccinated with PCV7. In a large cross-sectional study in 2013, vaccination uptake of the fourth booster dose was estimated at approximately 85%, but with a significant delay in timely administration of the booster [11]. In June 2019, the number of PCV primary doses was reduced, switching to a 2 + 1 schedule. For adults, pneumococcal vaccination has been included in the NIP since 2011, with the 20-valent vaccine (PCV20) being the recommended vaccine since February 2023. Data regarding the impact of PCV13 on pneumococcal disease incidence in Greece is sparse, as there is no mandatory IPD surveillance program except for pneumococcal meningitis.

Although the impact of PCV13 in Greece is difficult to establish, implementation of PCV13 into pediatric NIPs in other countries has greatly reduced vaccine serotype pneumococcal disease incidence, but disease caused by non-vaccine serotypes (NVTs) remains [9,12,13]. Two higher-valent PCVs were developed to combat the increase in disease caused by non-PCV13 serotypes: a 15-valent (PCV15), which includes PCV13 serotypes plus 22F and 33F, and a 20-valent (PCV20), which includes PCV15 serotypes plus 8, 10A, 11A, 12F, and 15B. In Greece, similar to other countries, these serotypes are increasing in prevalence primarily due to serotype replacement, as vaccine pressure removes competition and results in NVTs being able to colonize new hosts. A study conducted from November 2015 to December 2020 in Greek children aged ≤14 years with IPD and non-invasive disease (mainly otitis media) demonstrated PCV13, PCV15, and PCV20 serotypes caused 42.9%, 46.3%, and 64.9% of pneumococcal disease cases, respectively, over the entire study period [14]. A recent retrospective analysis of pneumococcal meningitis incidence in Greece observed a significant increase in the proportion of disease caused by NVTs across all ages following PCV13 introduction. From 2010 to 2012, 54.7% of cases were due to NVTs, compared to 77.2% from 2017 to 2019 [15]. Of the 18 fatal meningitis cases reported in the study, a large proportion of fatalities were caused by the additional serotypes included in higher-valent PCVs, with serotypes 10A, 8, and 22F contributing to 22% of all fatalities [15]. Finally, in a cross-sectional study from 2017 on pneumococcal carriage in children fully vaccinated with PCV13 in Greece, NVTs represented 83.8% of total isolates when excluding serogroups with mixed PCV13 and non-PCV13 serotypes [16]. Serotypes/serogroups 15A/B/C/F and 11A/D/E were the most prevalent serotypes/serogroups in pneumococcal carriage (13.8% and 11.4%, respectively). As such, vaccines that target additional serotypes are necessary to combat the increase in NVT disease and improve public health.

Over the period 2010 to 2020, PCV15 and PCV20 are estimated to cover 33.5% and 53.5% of meningitis cases in Greece, respectively [15]. Given the broader serotype coverage provided by higher-valent PCVs, PCV15 and PCV20 are anticipated to have an additional impact on disease incidence in both vaccinated and unvaccinated populations. Cost-effectiveness analyses (CEAs) of PCVs in Greece are sparse, and there are currently no published, peer-reviewed studies that estimate the cost-effectiveness of PCV15 or PCV20 in a pediatric Greek population. However, there is evidence that suggests higher-valent vaccines will result in greater public health and economic impact compared to lower-valent alternatives when included in the infant NIP. One previous analysis from Greece demonstrated the potential impact of PCV13 compared to PCV7 and PCV10 and found PCV13 to be cost-effective or cost-saving when compared to both lower-valent alternatives [17]. Additionally, a recent systematic review and meta-analysis of CEAs in high- and low-income countries found that PCV13 was consistently cost-effective compared to PCV10 when herd effects were considered [18]. In Greece, vaccinations are financially supported through a specific budget allocated by the Ministry of Finance and Ministry of Health, and vaccines included in the Greek NIP and the positive reimbursement list are fully reimbursed as per the NIP recommendations. As such, it is vital to assess the potential public health and economic impact of new, higher-valent vaccines in a Greek setting to ensure they are a worthwhile investment for the public. Therefore, the objective of this study is to estimate the clinical and economic impact of switching from PCV13 to PCV15 in 2023 compared with switching from PCV13 to PCV20 in 2024 for children in Greece.

## 2. Materials and Methods

### 2.1. Model Structure

A previously published decision-analytic model was adapted for the Greek setting to compare the clinical and economic impact of switching from PCV13 to PCV15 in 2023 or to PCV20 in 2024 (after the expected licensure in infants) over a 10-year time horizon from a payer perspective [19,20,21]. The vaccine switching model uses historic age- and serotype-specific IPD incidence data to predict prospective pneumococcal disease trends based on the observed retrospective data across all ages and serotypes. However, Greece does not have an active national, regional, or sentinel pneumococcal surveillance system to inform historic incidence rates. In lieu of local historic data, the model uses recent estimates of IPD serotype distribution in Greece and historical IPD incidence data from the US to project future incidence and serotype distribution changes under each pediatric PCV NIP strategy. Future IPD incidence for newly covered serotypes contained in PCV15 or PCV20 is projected using the annual relative reduction observed after PCV13 implementation in the US [9]. Historical trends from the US were used because of similarities in dosing schedules in Greece as of 2019 (3 + 1), uptake, program implementation, and catch-up programs for those aged less than 60 months both for PCV7 and for PCV13. Additionally, in a Greek study on adult hospitalized community-acquired pneumonia (CAP), the proportions of pneumococcus and PCV13 serotypes were comparable to those reported in a US CAP study but substantially lower than those reported in a Spanish study and a UK study, indicating similarities in pneumococcal epidemiology between Greece and the US [22]. Moreover, previous infant PCV modeling studies in Greece have used US epidemiological data for estimating future higher-valent vaccine effects [17]. Disease trends are projected across the entire population to capture both the direct and indirect effects of vaccination. By using real-world evidence and historical trends in the incidence of covered and non-covered serotypes, the model captures both vaccine pressure on covered serotypes and the replacement of NVTs, as NVTs are modeled to increase or decrease in incidence based on their previously observed real-world behavior after implementation of a pediatric PCV13 NIP.

Following the implementation of nonpharmaceutical interventions to reduce COVID-19 transmission (i.e., restrictions on large gatherings, masking, and business closures), reductions in the incidence of IPD were observed in some countries. Yet, recent data from multiple European countries demonstrates that IPD incidence has rebounded or exceeded pre-COVID-19 pandemic levels after social distancing measures were lifted, and serotype distributions remain the same [23,24,25]. As a result, the model assumes current pneumococcal disease incidence rates are similar to those in 2019 and models prospectively beginning in 2023.

### 2.2. Population

The Greek population size was obtained from the Hellenic Statistical Authority and stratified into seven age groups (0–<2, 2–4, 5–17, 18–34, 35–49, 50–64, and 65+) [26]. The model assumed a pediatric vaccination coverage rate of 84.5% [11].

### 2.3. Base-Case Cost-Effectiveness Analysis

The base-case cost-effectiveness analysis estimates the number of disease cases and deaths over a 10-year time horizon from a third-party payer perspective. Costs and outcomes are discounted at a rate of 3.5%. Historical IPD incidence trends from the US are used to project future incidence and serotype behavior. Epidemiological, economic, and population inputs used in the base-case analysis can be found in Table 1.

### 2.4. Invasive Pneumococcal Disease Incidence

IPD is defined in the model as bacteremia and meningitis. Age-specific IPD incidence was derived by extrapolation based on recent estimates of pneumococcal meningitis incidence in Greece as published by Xirogianni et al., 2022 [15]. The average meningitis incidence for each age group from 2010 to2020 was divided by the proportion of IPD known to be meningitis. Based on available data, we assumed 10.2% of IPD presented as meningitis in children aged 0 to 17 years and 7.0% of IPD presented as meningitis in the population aged 17 years and up [14,28]. For simplicity, the IPD incidence rate for individuals aged 5–17 was calculated as a simple average of the calculated incidence for 5–9-, 10–14-, and 15–19-year-olds.

IPD incidence was modeled for the following serotype groups: PCV7 covered (serotypes 4, 6B, 9V, 14, 18C, 19F, and 23F), incremental PCV10 (serotypes 1, 5, and 7F), incremental PCV13 (serotypes 3, 6A, and 19A), incremental PCV15 (serotypes 22F and 33F), incremental PCV20 (serotypes 8, 10A, 11A, 12F, and 15B), and not covered NVTs. The IPD serotype distribution for children under 18 years was sourced from a prospective research study conducted between November 2015 and December 2020 in Greece [14]. Due to the small sample size, the model used pooled serotype data from the five years of data collection to inform current serotype dynamics. The serotype distribution for individuals over the age of 18 years was sourced from Xirogianni et al., 2022 [15]. Due to the small number of isolates collected in the study over 10 years, the serotype distribution from 2010–2020 was pooled to estimate the serotype distribution for 2019.

Future IPD incidence rates for modeled serotype groups were forecasted based on observed US incidence trends. Regression models were independently fitted to historical data from covered (i.e., after PCV13 introduction) and non-covered (i.e., before PCV13 introduction) periods. Based on R-squared values reflecting best fit, linear or logarithmic functions are used to forecast non-covered serotypes to minimize unrealistic increases in disease incidence, while linear, logarithmic, exponential, or power functions are used to forecast covered serotypes. Therefore, assuming a switch to PCV15 (2023) or PCV20 (2024), the IPD incidence rates for newly covered serotypes in higher-valent PCVs were forecasted based on covered periods (i.e., incidence reductions after PCV13 implementation). Scenario analyses were also run given the uncertainty of higher-valent vaccines direct and indirect effects.

### 2.5. Non-Invasive Pneumococcal Disease Incidence

Non-invasive pneumococcal disease is defined in the model as pneumococcal pneumonia and otitis media (OM). However, the etiology of pneumonia and OM is largely unknown, as cultures are rarely performed, leaving few estimates of pneumococcal pneumonia and OM incidence. Global estimates indicate that the proportion of non-invasive pneumococcal disease varies and can range between 20 and 50% of all-cause pneumonia and OM [39,40]. As such, the model uses incidence rates for all-cause pneumonia and OM and conservatively assumes that 20% of all pneumonia and OM are caused by *S. pneumoniae*. Future non-invasive disease incidence rates are assumed to vary proportionally with IPD, given that serotype distribution is unknown for non-invasive disease. This assumption means that vaccination programs will impact the incidence and serotype distribution of IPD and non-invasive diseases similarly.

Due to a lack of local incidence data for all-cause pneumonia and OM, the model used the most recently available US incidence data, as reported in a recent cost-effectiveness analysis presented to the US Centers for Disease Control and Prevention (CDC) [27].

### 2.6. Mortality

All-cause mortality rates per 100,000 for all age groups were calculated using data from the Hellenic Statistical Authority [41]. Case-fatality rates (CFR) for IPD were derived from the US CDC Active Bacterial Core Surveillance System (ABCs) 2019 data and were assumed to be the same for meningitis and bacteremia [28]. A retrospective cohort study of a US claim database was used to derive CFRs for hospitalized pneumonia in those over 18 years of age [31]. For individuals under the age of 18, CFR was derived from the CDC cost-effectiveness report for PCV15 [29,30]. Consistent with epidemiologic data and previous CEAs, outpatient pneumonia and OM have no risk of fatality.

### 2.7. Economic Inputs

Vaccination acquisition costs were obtained from the latest available official Greek Price Bulletin at the time of the analysis, in which the retail prices of PCV13, PCV15, and PCV20 were €63.07, €72.32, and €70.98, respectively [32]. Age-specific, direct medical costs associated with each case of bacteremia, meningitis, hospitalized and non-hospitalized pneumonia, and OM were compiled from Naoum et al., 2020, and official sources such as Diagnosis Related Group tariffs [33,34]. Costs from Naoum et al., 2020, were inflated to 2023 euros.

### 2.8. Utility Parameters

Age-specific baseline utility values for healthy individuals were sourced from Greek EQ-5D data as published by Kontodimopoulos et al., 2008 and adjusted to fit the age groups in the model [35]. Because there are no studies assessing the utility of individuals under the age of 18 in Greece, the assumed utility for this age group is the same as the reported utility for 18–24-year-olds. A previous CEA for PCVs was used to inform utility decrements for each episode of pneumococcal disease [42]. Utility decrements of 0.0079, 0.0232, 0.004, 0.006, and 0.005 were used for bacteremia, meningitis, non-hospitalized pneumonia, hospitalized pneumonia, and OM, respectively [36,37,38].

### 2.9. Scenario and Sensitivity Analyses

Scenario analyses were conducted to test the uncertainty of certain input parameters. The proportion of all-cause non-invasive disease caused by *S. pneumoniae* varied by +/−10%, the discount rate varied from 0% to 5%, and the time horizon was shortened to 5 years. To project reductions in incidence for newly covered serotypes, we conducted analyses using annual reductions in IPD after PCV13 pediatric NIP implementation observed in Canada, the United Kingdom (UK), and Israel [9]. Additionally, we tested a scenario in which historical trend lines from the UK were used to project the future incidence of PCV13 serotypes.

A probabilistic sensitivity analysis (PSA) was conducted to assess the variance of ICER when multiple inputs were varied at the same time. Parameters that varied in the PSA included IPD incidence, percentage of IPD causing meningitis, percentage of non-invasive disease caused by *S. pneumoniae,* baseline utility values, utility decrements, and direct medical cost per episode of disease. A Monte Carlo simulation with 1000 iterations was run, and incremental costs and QALYs from each iteration were compiled in a scattered plot.

## 3. Results

### 3.1. Public Health Impact

**Figure 1:**
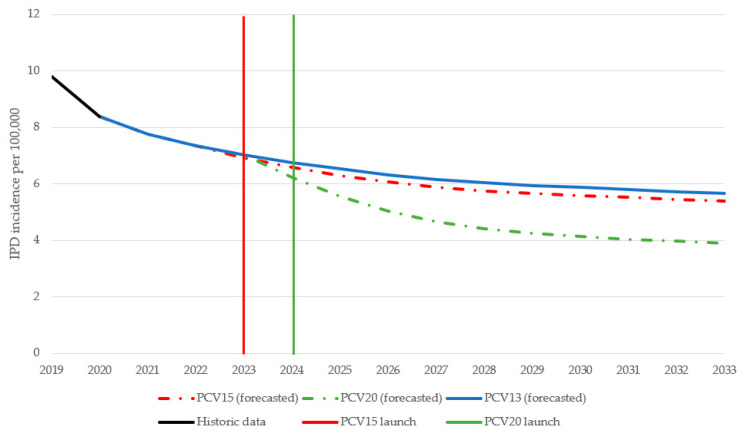
Forecasted IPD incidence in children aged <2 years over time for each vaccination strategy. PCV13 = 13-valent pneumococcal conjugate vaccine; PCV15 = 15-valent pneumococcal conjugate vaccine; PCV20 = 20-valent pneumococcal conjugate vaccine.

**Figure 2:**
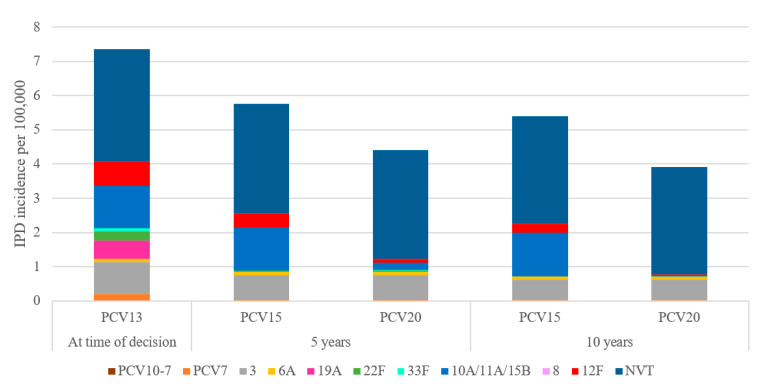
Forecasted serotype distribution for children aged under 2 years old after 10 years under each vaccination strategy. PCV15 = 15-valent pneumococcal conjugate vaccine, PCV20 = 20-valent pneumococcal conjugate vaccine, NVT = non-vaccine type.

Over 10 years, switching to a higher-valent vaccine is expected to result in greater reductions in overall IPD incidence in children under 2 years old compared to maintaining vaccination with PCV13 (Figure 1). IPD incidence in children under 2 was estimated to decrease by 25.9% under a PCV15 program compared to a 46% decrease under a PCV20 program. The greater reduction in IPD incidence under a PCV20 program was driven by a decrease in the incremental serotypes included in PCV20 that are not included in PCV15, particularly serotypes 10A, 11A, and 15B (Figure 2). Compared to PCV15, switching from PCV13 to PCV20 in 2024 was estimated to avert an additional 250 IPD cases, 54,876 otitis media cases, 8458 pneumonia cases, and 255 deaths over 10 years across all ages (Table 2).

### 3.2. Economic Impact

Over 10 years, PCV20 is estimated to reduce vaccine acquisition costs and direct medical costs by over EUR 4.5 million and EUR 53.5 million, respectively (Table 2). In total, switching from PCV13 to PCV20 in 2024 could save the Greek taxpayer an additional EUR 58.1 million compared to switching from PCV13 to PCV15 in 2023.

### 3.3. Base-Case Cost-Effectiveness Results

Translated to life years (LYs) and quality-adjusted life years (QALYs), PCV20 was estimated to save an additional 551 LYs and 486 QALYs compared to PCV15 (Table 2). Even when implemented one year later than PCV15, PCV20 is the dominant cost-saving strategy compared to PCV15 and could save the public payer over EUR 110,000 per QALY gained.

### 3.4. Scenario and Sensitivity Analyses

The cost-effectiveness results of each scenario analysis were consistent with the base case, in which PCV20 was the dominant strategy, leading to better clinical outcomes and lower total costs compared to PCV15 (Table 3). Using trend lines from different countries to predict future incidence of newly covered serotypes under a PCV15 or PCV20 program, PCV20 was associated with total cost savings ranging from EUR 45.6 to EUR 57.0 million and 376 to 467 more QALYs. When alternate discount rates were used, the total cost savings ranged from EUR 50.5 to EUR 81.5 million, and incremental QALYs ranged from 386 to 879. Varying the proportion of all-cause non-invasive disease due to *S. pneumoniae* resulted in a range of total cost savings from EUR 47.5 to EUR 68.7 million and 388 to 585 more QALYs. Reducing the time horizon to 5-years led to total cost savings of EUR 18.2 million and 93 more QALYS. Using trend lines from the UK to predict future incidence of PCV13-type disease resulted in the greatest incremental costs and QALYs, with total cost savings of EUR 116.1 million and 1004 QALYs saved.

**Figure 3:**
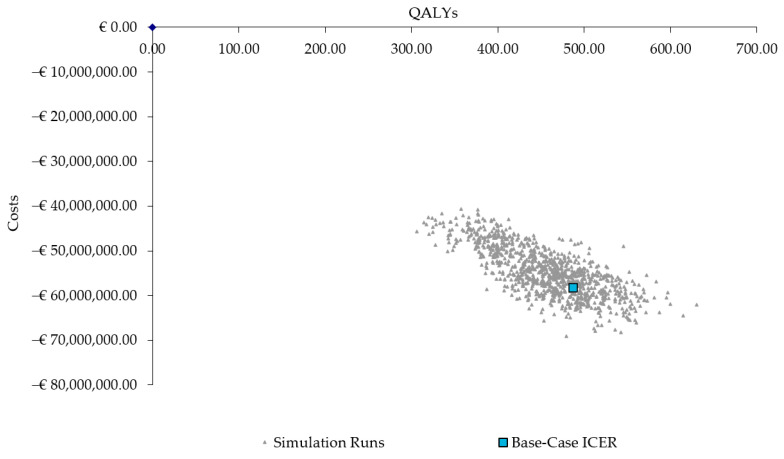
Scatter plot of probabilistic sensitivity analysis results. QALYs = quality-adjusted life years; ICER = incremental cost-effectiveness ratio.

The results from the PSA also confirmed the robustness of the base-case results, with 100% of the ICERs in the fourth quadrant, indicating the PCV20 strategy consistently results in more QALYs and lower costs (Figure 3).

## 4. Discussion

The present study used a decision-analytic model to forecast the economic and public health impact of switching from PCV13 to PCV15 in 2023 compared with switching to PCV20 in 2024 in Greece’s pediatric NIP. The economic evaluation estimated that switching to a higher-valent vaccine would have a significant positive public health impact and provide cost savings by reducing disease incidence across all ages. Compared to switching to PCV15, switching from PCV13 to PCV20 in 2024 was expected to generate a greater reduction in pneumococcal disease cases and deaths, primarily due to the prevention of disease caused by serotypes 10A, 11A, and 15B. Therefore, PCV20 is estimated to be cost-saving for the Greek public payer, even if implemented one year later than PCV15. Both the PSA and scenario analyses corroborated the base-case results, which were found to be relatively insensitive to variation in time horizon, input parameters, higher-valent vaccine effects, and model assumptions, likely because of the large amount of current circulating PCV20-unique serotypes (i.e., PCV20 remained dominant over PCV15).

At the time of writing, there are no peer-reviewed manuscripts that assess the cost-effectiveness of switching from PCV13 to PCV15 compared to switching from PCV13 to PCV20 in a pediatric NIP. However, our results are aligned with two published studies, one from South Africa and another from the Netherlands, that estimated the public health impact of switching from a 10-valent or 13-valent PCV to higher-valent alternatives. In South Africa, switching from PCV13 to PCV20 in 2025 was estimated to avert more pneumococcal disease cases and deaths compared to all other vaccination strategies, which included switching from PCV13 to either two 10-valent alternatives or PCV15 [20]. In the Netherlands, which has a PCV10 NIP, switching to PCV13 followed by a subsequent switch to PCV20 was expected to generate the largest decrease in pneumococcal disease cases and deaths compared to maintaining PCV10 vaccination and then switching to PCV15, even when PCV20 is implemented one year later than PCV15 [19].

The prevalence of NVT IPD has been increasing following the introduction of PCVs into infant NIPs. Across Europe, the proportion of disease caused by PCV20 serotypes is increasing. A recent publication estimated that PCV20-unique serotypes 8, 10A, 11A, 12F, and 15B accounted for 29.3% of IPD cases in Europe across all ages in 2018, while PCV15-unique serotypes 22F and 33F only contributed to 6.6% and 2.5% of cases, respectively [43]. Countries such as France and Belgium have also reported a much higher incidence of IPD in children caused by PCV20-unique serotypes compared to PCV15-unique serotypes [43,44]. As such, the broader serotype coverage of PCV20 compared to PCV15 is expected to substantially reduce the clinical and economic burden associated with pediatric pneumococcal disease.

The limitations of our analysis are primarily related to data availability for informing model inputs. First, because Greece does not have mandatory IPD surveillance except for pneumococcal meningitis, IPD incidence data for the analysis start year had to be extrapolated based on recently published incidence data for pneumococcal meningitis. Second, to project the incidence of PCV13 serotypes, the model relied on historical serotype trends in PCV13-type disease incidence from the US due to a lack of historical Greek data. As such, this resulted in the model estimating a complete disappearance of 19A incidence after 5 years, which is uncertain based on the current epidemiology in Greece. To address this, a scenario was conducted in which historical trend lines from the UK were used to project future PCV13-type disease. In this scenario, 19A incidence persisted over the 10-year time horizon, yet PCV20 remained the cost-saving option compared to PCV15. Third, there are no data available regarding non-invasive pneumococcal disease incidence in Greece, so the model uses US data on incidence rates, considering the US and Greece have similarities in their PCV13 vaccination policies and implementation (including dosing schedules, catch-up recommendations, vaccination uptake for children, and vaccination recommendations for adults). Fourth, lacking age-specific utility values for individuals less than 18 years old, the model assumed that the utility value for 18–24-year-olds as reported by Kontodimopoulous et al., 2008 was applicable to pediatric age groups. This assumption was tested in the PSA, and the results remained consistent with the base case. Additionally, the utility decrements for each disease state come from a variety of different populations; however, we assumed them to be applicable for all age groups, as done in a previous CEA by Melegaro and Edmunds [42]. These parameters were included in the PSA, and PCV20 remained cost-saving. Fifth, the model assumed that 20% of all non-invasive diseases were caused by *S. pneumoniae,* as the true proportion is largely unknown. However, this assumption varied in scenario analyses, and results remained consistent. Sixth, because there is no data regarding the impact of higher-valent vaccines, the model had to make assumptions regarding disease reduction for newly covered serotypes, which may not be representative of their effectiveness in the real world. Future cost-effectiveness analyses of higher-valent vaccines would greatly benefit from real-world evidence demonstrating their impact. Seventh, considering Greek providers can choose whether to vaccinate infants with PCV13 or PCV15, the model structure and design do not allow for mixed standards of care as a comparator. Finally, the analysis conservatively assumed a third-party payer perspective and does not account for any indirect non-medical costs, such as those related to caregiver productivity loss, transportation, or lost leisure time, which could better reflect the overall benefits of switching to PCV20 if included.

Despite these limitations, the current study applies a well-established model structure to evaluate the cost-effectiveness of switching from PCV13 to either PCV15 or PCV20 in the Greek infant NIP. It is important to note that the reported results only capture the impact of pediatric vaccination, and further reductions in IPD and non-invasive disease may be possible if elderly or at-risk populations in Greece were also vaccinated with PCV20. Considering the current epidemiology in Greece, PCV20 could offer substantial public health and economic benefits in Greece compared to PCV15. Additionally, even if the price of PCV20 were increased by 45.5%, PCV20 would still be a cost-saving option compared to PCV15. Therefore, policymakers in Greece could consider waiting until PCV20 is licensed for pediatric use before making decisions regarding which higher-valent vaccine should replace PCV13. The model incorporated the best available evidence from the Greek setting, but future analyses would greatly benefit from more comprehensive local epidemiological data to validate PCV20′s dominance over PCV15. Regardless, the results were tested with both a PSA and various scenario analyses, demonstrating the cost-saving results for PCV20 are robust under a range of alternative assumptions and model inputs.

## 5. Conclusions

The present study showed that the use of PCV20 in the Greek NIP could reduce pneumococcal disease cases and provide the greatest benefit to population health in comparison to PCV15. Additionally, the Greek public payer could save substantial health care costs due to the higher number of disease cases prevented. The results of this study are important for health policy makers to make informed decisions about the allocation of limited healthcare resources and to prioritize health interventions that provide the most value for the Greek healthcare system.

## Figures and Tables

**Table 1:** Population, clinical, and economic parameters used in the base-case analysis.

Parameter	Age Group, Years						
	0–<2	2–4	5–17	18–34	35–49	50–64	65+
Population ^a^	214,274	322,969	1,352,882	2,405,762	2,393,924	2,017,623	2,108,852
IPD incidence per 100,000 ^b^	9.8	9.8	1.7	3.1	4.6	6.1	6.3
Inpatient CAP incidence per 100,000 ^c^	927	293	94	123	123	4769	1477
Outpatient CAP incidence per 100,000 ^c^	2.891	3268	1245	623	623	1104	2864
Mild OM incidence per 100,000 ^c^	63,494	38,974	11,765				
Percentage of IPD that is meningitis ^c,d^	10.2%	10.2%	10.2%	7.00%	7.00%	7.00%	7.00%
Case fatality rates							
IPD ^e^	0.060	0.019	0.064	0.027	0.107	0.139	0.110
Inpatient CAP ^f^	0.009	0.004	0.013	0.014	0.014	0.038	0.080
Vaccine costs, per dose (€) ^g^							
PCV13	63.07						
PCV15	72.32						
PCV20	70.98						
Direct costs (€)							
Bacteremia ^h^	3685	3685	3685	3685	3685	3685	3685
Meningitis ^h^	3762	3762	3762	3762	3762	3762	3762
Inpatient pneumonia ^i^	8641	8641	8641	8641	8641	8641	8641
Outpatient pneumonia ^i^	130	130	130	130	130	130	130
OM ^h^	542	542	542	542	542	542	542
Utility weights							
Baseline utility ^j^	0.94	0.94	0.94	0.93	0.82	0.69	0.65
Disutility of bacteremia ^k^	0.0079	0.0079	0.0079	0.0079	0.0079	0.0079	0.0079
Disutility of meningitis ^k^	0.0232	0.0232	0.0232	0.0232	0.0232	0.0232	0.0232
Disutility of inpatient CAP ^k,l^	0.006	0.006	0.006	0.006	0.006	0.006	0.006
Disutility of outpatient CAP ^l^	0.004	0.004	0.004	0.004	0.004	0.004	0.004
Disutility of mild OM ^m^	0.005	0.005	0.005				

^a^ Hellenic Statistical Authority [26]; ^b^ Xirogianni et al. adjusted by proportion of IPD presenting as meningitis [15]; ^c^ Ayabina et al. [27]; ^d^ Koutouzis et al. [14]; ^e^ US CDC 2019 ABCs [28]; ^f^ Data for individuals less than 18 years of age from Stoecker et al. and Kobayashi et al., data for individuals older than 18 years of age from Averin et al. [29,30,31]; ^g^ Greek Price Bulletin [32]; ^h^ Diagnosis Related Group tariffs [33]; ^i^ Naoum et al. inflated to 2023 EUR [34]; ^j^ Kontodimopoulos et al. [35]; ^k^ Bennett et al. [36]. ^l^ Vold Pepper and Owens [37]; ^m^ Oh et al. [38].

**Table 2:** Base-case results on public health impact, economic impact, and cost-effectiveness of switching from PCV13 to PCV15 or to PCV20 in Greece’s pediatric NIP.

Outcomes *	PCV15	PCV20	Incremental
Disease cases			
Bacteremia	4056	3832	−224
Meningitis	461	435	−26
Inpatient pneumonia	100,462	95,289	−5173
Outpatient pneumonia	388,396	385,111	−3285
OM **	742,626	687,749	−54,876
Total	1,236,000	1,172,416	−63,584
Deaths			
IPD	444	426	−18
Inpatient pneumonia	5920	5683	−237
Total	6364	6109	−255
Costs (€)			
Vaccine	145,867,540	141,300,714	−4,566,825
IPD	12,534,179	11,885,388	−648,791
Pneumonia	688,347,884	656,701,892	−31,645,992
OM	301,982,740	280,705,929	−21,276,811
Total	1,148,732,343	1,090,593,924	−58,138,419
Outcomes			
Life years	78,959,352	78,959,902	551
QALYs	48,420,807	48,421,293	486
ICER			PCV20 dominant (cost-saving)

Abbreviations: ICER = incremental cost-effectiveness ratio; IPD = invasive pneumococcal disease; NIP = national immunization program; OM = otitis media; PCV = pneumococcal conjugate vaccine; QALY = quality-adjusted life year. Note: The cost-effectiveness analysis was run for two scenarios over a 10-year time horizon from a payer perspective: (1) switching from PCV13 to PCV15 in 2023 and (2) switching from PCV13 to PCV20 in 2024. * All outcomes are pneumococcal-disease outcomes. ** Impact on OM only considered for children aged 0–17 years old.

**Table 3:** Cost-effectiveness results from scenario analyses.

	Incremental Cost	Incremental QALYs	ICER
Base-case results	−58,138,419	486	PCV20 cost-saving
Time horizon			
5-year	−18,203,282	93	PCV20 cost-saving
Other country trend lines to predict future PCV20-13 incidence			
Canada	−45,673,006	376	PCV20 cost-saving
Israel	−57,043,452	467	PCV20 cost-saving
UK	−51,827,542	418	PCV20 cost-saving
Discount rates			
0%	−81,503,059	879	PCV20 cost-saving
5%	−50,573,641	386	PCV20 cost-saving
Proportion of all-cause non-invasive disease due to *S. pneumoniae*			
10%	−47,500,013	388	PCV20 cost-saving
30%	−68,776,873	585	PCV20 cost-saving
Other country trend lines to predict future PCV13 incidence			
UK	−116,193,893	1004	PCV20 cost-saving

Abbreviations: QALYS = quality-adjusted life-years; ICER = incremental cost-effectiveness ratio; UK = United Kingdom; PCV = pneumococcal conjugate vaccine.

## Data Availability

All input data for the model are available in the tables published in this manuscript.
